# Therapeutic Targeting of Alternative Splicing: A New Frontier in Cancer Treatment

**DOI:** 10.3389/fonc.2022.868664

**Published:** 2022-04-08

**Authors:** Anthony J. Murphy, Alex H. Li, Peichao Li, Hong Sun

**Affiliations:** ^1^ Department of Environmental Medicine, New York University School of Medicine, New York, NY, United States; ^2^ Department of Thoracic Surgery, The Second Hospital, Cheeloo College of Medicine, Shandong University, Jinan, China

**Keywords:** RNA splicing, alternative splicing, cancer therapeutics, splicing factors, spliceosome inhibitors

## Abstract

The ability for cells to harness alternative splicing enables them to diversify their proteome in order to carry out complex biological functions and adapt to external and internal stimuli. The spliceosome is the multiprotein-RNA complex charged with the intricate task of alternative splicing. Aberrant splicing can arise from abnormal spliceosomes or splicing factors and drive cancer development and progression. This review will provide an overview of the alternative splicing process and aberrant splicing in cancer, with a focus on serine/arginine-rich (SR) proteins and their recently reported roles in cancer development and progression and beyond. Recent mapping of the spliceosome, its associated splicing factors, and their relationship to cancer have opened the door to novel therapeutic approaches that capitalize on the widespread influence of alternative splicing. We conclude by discussing small molecule inhibitors of the spliceosome that have been identified in an evolving era of cancer treatment.

## Introduction

Splicing, the process of converting precursor mRNA (pre-mRNA) into mature mRNA *via* a system of highly specific and stepwise interactions, is considered a means of transcriptional diversification (1). Alternative splicing (AS) is pivotal in many aspects of cellular homeostasis along with cell differentiation and tissue and organ development (2). Maturely spliced mRNA variants can diversify the proteome by providing distinct characteristics to protein isoforms that permit dynamic cellular function. However, in the event that the system (spliceosome complex and splicing factors) is hostilely reconfigured, as in the case with many cancers, splicing also possesses an inherent risk of generating malignant protein isoforms (3).

There are many RNA-protein complexes and associated regulatory proteins with which endogenous or exogenous factors can interfere and threaten the integrity of splicing. Many of these components have been determined to cause global changes when dysregulated and contribute to the initiation and progression in human cancers (4). The phenomenon of dysregulated splicing in cancer has recently been recognized to occur frequently across all cancer types (5) and to impact all seven groups of mechanisms of anticancer drug resistance (6). Due to this recent recognition, there has been an increase in attention to understanding the splicing process, components involved, and their susceptibility to pharmacological intervention. The pursuit of targeting splicing for therapeutic purposes has yielded unique and selective compounds and novel therapies. Given the prevalence of therapeutic resistance across cancer types and an unmet medical need for targeted therapies for these patients, it is a priority to investigate novel molecular mechanisms that may reveal druggable targets and new therapeutic strategies.

## Spliceosome and Splicing Factors

**Figure 1:**
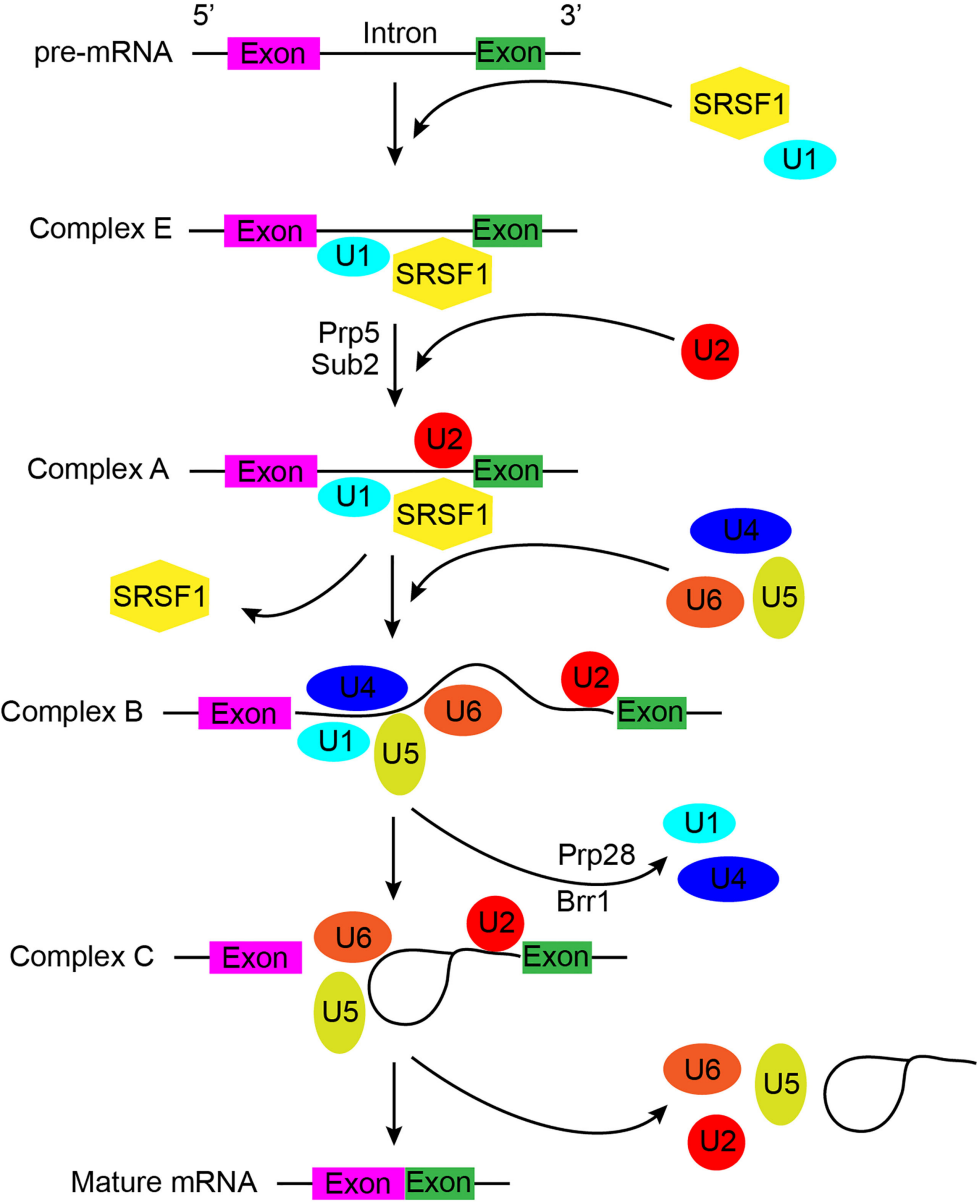
A general schematic of pre-mRNA splicing by the major spliceosome, which describes the process in detail and indicates possible mechanisms underlying the potential function of spliceosome during splicing. Colored cuboids indicate exons, while the solid black lines represent introns.

RNA splicing is orchestrated by a flexible and dynamic macromolecular machine, known as the major spliceosome (hereby referred to as the spliceosome). The spliceosome comprises of more than 300 proteins including more than 100 non-core or accessory regulatory proteins and preferentially process U2-type introns (7). At the core of the spliceosome lies U1, U2, U4, U5, and U6 small nuclear ribonucleoproteins (snRNPs), seven Sm proteins or Lsm (U6-specific) proteins, and additional snRNP-specific factors (8). Each core snRNP also contains a small nuclear RNA (snRNA), which facilitates RNA-RNA and RNA-protein interactions during the highly dynamic splicing process. The array of spliceosome components assembles in distinct manners throughout the splicing process; termed complexes E, A, pre-B, B, B^act^, B*, C, C*, P, and ILS (**Figure 1**) (8, 9). In addition, the minor spliceosome, responsible for processing less than 1% of human splice sites, differs from the major spliceosome in structurally containing four distinct snRNPs (U11, U12, U4atac, and U6atac), and preferentially bound to U12-type introns (10).

**Figure 2:**
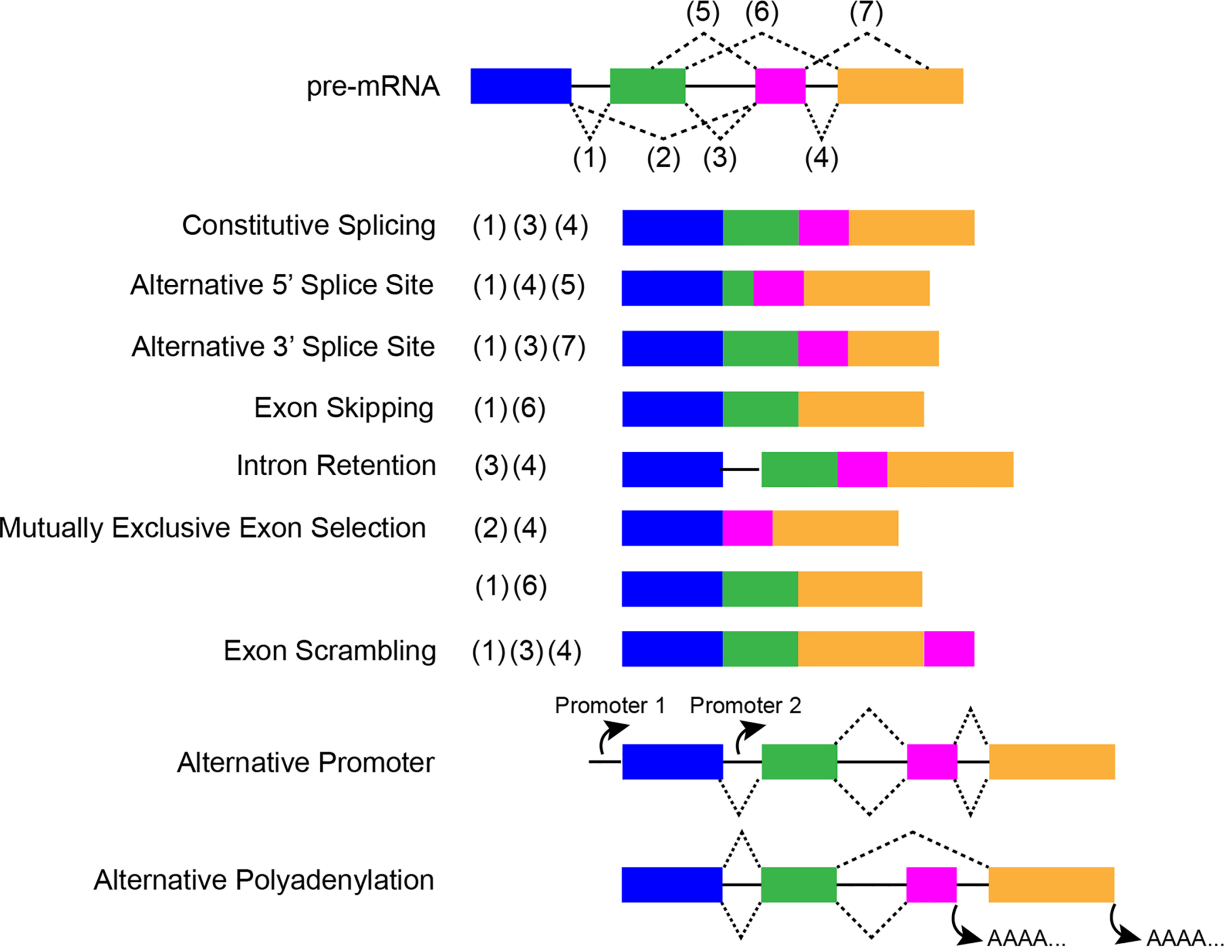
A schematic about constitutive and ASEs, which displays the special characteristics of each event. Colored cuboids represent exons, while the solid black lines indicate introns. Dotted lines indicate splicing sites.

Alternative splicing (AS) generates various forms of mature mRNA through multiple mechanisms including: alternative 3’ or 5’ splice sites, exon skipping, mutually exclusive exon selection, intron retention, exon scrambling, alternative promoter, and alternative polyadenylation (**Figure 2**) (11). Successful and accurate AS requires both *cis*-acting elements *and trans*-acting factors. The serine/arginine rich splicing factors (SRs) are important *trans*-acting proteins involved in every step of the recruitment and assembly of the spliceosome (12). The twelve members (SRSF1-12) are characterized by having one or two RNA recognition motifs (RRM) in the N-terminus and a domain rich in arginine and serine in the C-terminus (RS domain) that mediate many protein-protein and protein-RNA interactions. These proteins function by binding *cis*-regulatory elements within pre-mRNA *via* their RRM, recruiting the spliceosomes and associated proteins, and regulating splice-site selection (13, 14). Another major family of splicing factors is the heterogeneous nuclear ribonucleoprotein (hnRNP) family. Canonical hnRNPs contain an RRM domain, RGG boxes, and additional domains that promote functional diversity by fostering a variety of protein-protein interactions (15). hnRNPs have been found to repress spliceosome assembly by binding to exonic splicing silencer (ESS) elements to block exon recognition (16).

## Alternative Splicing and Cancer

In the past decade, accumulating evidence demonstrated the important roles of aberrant RNA splicing in tumor initiation and progression. An analysis of The Cancer Genome Atlas (TCGA) data from 33 different cancer types suggested the mutations in 119 splicing factor genes as putative cancer drivers, which consisted more than half of all proteins in splicing machinery (17). In addition to gene mutation, more than 70% of splicing factors and 84% of RNA-binding proteins have been found to be dysregulated at mRNA levels in cancers (18, 19). Perturbation of splicing machinery and regulatory factors *via* mutations or alterations lead to dysregulation of alternative splicing events (ASEs) and cancer-specific isoforms of oncogenes and tumor suppressors, which contribute to every hallmark of cancer [reviewed in (3, 5)].

### Aberrant Splicing in Cancer

Dysregulation of AS in cancer has been shown to impact potent oncogenes and tumor suppressors. A comprehensive analysis of 8,705 patients across 32 cancer types from (TCGA) revealed that tumors display up to 30% more ASEs compared to matched normal tissues (20). Exon skipping and alterative 3’ splice sites were significantly increased in tumors (20). Aberrant ASEs have been documented in all classical hallmarks of cancer including: resisting cell death (e.g. BCL2L1), sustaining proliferative signaling (e.g. CD44), evading growth suppressors (e.g. p53), invasion and metastasis (e.g. Ron), avoiding immune destruction (e.g. CD45), immortality (e.g. hTERT), angiogenesis (e.g. VEGF), and deregulation of cellular energetics (e.g. PKM) (21). Moreover, upregulation of cancer-specific isoforms of TP73, CDH17, KLF6, FGFr2, FGFR3, DNMT3b3, and OPN has been reported in human hepatocellular carcinoma (HCC) and promoted cell cycle progression, proliferation, invasion and metastasis (22).

In contrast to driver mutations that affect well-known oncogenes such as Ras, synonymous or “silent” mutations frequently contribute to cancer and are often associated with changes in splicing (23). Synonymous mutations in oncogenes were determined to impact key motifs utilized by splicing factors to generate alternatively spliced transcripts (23). In tumor suppressors, single nucleotide variants causing intron retention were found to be enriched, the vast majority of which generated a premature termination codon (PTC), leading to loss of function *via* nonsense-mediated mRNA decay (NMD) or truncated proteins (24).

The most common mode of AS is exon skipping; however, when analyzing different modes of AS using transcriptome data from TCGA, Dvinge and Bradley (2015) found no obvious biases in 5’ or 3’ splice site recognition or exon skipping relative to normal control samples (25). Instead, it was reported that almost all analyzed cancer types showed increased levels of intron retention, with the exception of breast cancer, whereby this observation was reversed; intron retention characterized normal breast tissue rather than cancer tissues (25). A different group showed that exon skipping as well as intron retention were predominant splicing events in breast cancer (26). The discrepancy in reported intron retention in breast cancers was further explored in 50 tumorigenic and six non-tumorigenic breast cell lines, which did not show a consistent association between tumorigenic/non-tumorigenic status and degree of intron retention (25). Tissue- and cell-type specific splicing may confound the assessment of significant differences between tumors and normal samples across cancer types. While recent efforts have focused on determining transcriptome-wide changes in AS, the vast majority of studies that have explored cancer-associated changes in AS focus on specific aberrant splicing events of well-known oncogenes or tumor suppressors.

An example of specific ASEs of well-known oncogenes can be seen in the complex phenomenon of tumor heterogeneity. Tumor heterogeneity has been proposed to arise from clonal evolution, in which random mutations drive adaptation and selection. In addition, the phenomenon of tumor heterogeneity has been associated with the acquisition of cancer stem cell (CSC)-like properties, through which a subset of cancer cells obtains the capacity for self-renewal and thus are able to initiate and maintain tumor growth (27). With respect to the CSC model of tumor heterogeneity, it has been demonstrated that AS of Id1 generates two isoforms, Id1a and Id1b. Overexpression of the Id1b isoform resulted in upregulation of cancer stem cell markers ALDH1A1, Notch-1, Oct-4, Sox-2, Tert, and Sca-1 (28). Moreover, Id1b overexpression promoted self-renewal and adoption of CSC-like properties including generation of secondary tumorspheres (28).

The tumor microenvironment (TME) is another significant driver of tumor biology and includes noncellular and cellular components that assist in cancer progression. Noncellular components include, but are not limited, to growth factors, cytokines, RNA, DNA, metabolites, structural matrix, and matricellular proteins (29). Cellular components have been described as including fibroblasts and myofibroblasts, neuroendocrine cells, adipose cells, immune and inflammatory cells, the blood and lymphatic vascular networks, and extracellular matrix (ECM) (30). Hypoxia, an important noncellular component of the TME, has been shown to induce a splice switch in Fas, resulting in the exclusion of exon 6 in Fas pre-mRNA in human colorectal cancer cells (31). Exon 6 exclusion in Fas generates a soluble anti-apoptotic isoform of Fas, which may partly link hypoxia signaling and AS to the cellular ability to overcome apoptosis and survive under reduced oxygen tension (31).

While the knowledge of specific aberrant splicing events impacting well-established oncogenes or tumor suppressors is critical in understanding the impact that splicing has on cancer development and progression, the overwhelming observations in aberrant splicing points toward an upstream regulatory mechanism that may dictate widespread changes in the AS landscape. Splicing factors such as RNA binding proteins (RBPs) have been shown to participate in extensive autoregulatory feedback and cross-talk (32). Moreover, RBPs such as SRSF1 have been shown to be a direct target of oncogenes, providing a scenario whereby oncogenic activation of these splicing factors may lead to far-reaching alterations in splicing profiles across cancer types (33).

### SR Proteins and Cancer

Nearly all SR proteins known to date have been associated with cancer to some degree, many of which display relationships with common oncogenes and tumor suppressors. SRSF1 was the first SR protein to be linked to splicing, and since then, SRSF1 has been shown to play a role in mRNA transcription, RNA stability and nuclear export, NMD, translation, protein sumoylation (34). More importantly, SRSF1 was also the first SR protein suggested to act as a proto-oncogene to promote tumorigenesis and is overexpressed in a number of cancers (35) [reviewed in (34)]. Of note, Pio et al. used genome-wide microarrays and an algorithm that uses data from exon and junction probes in order to identify a network of splicing events under control of SRSF1 in lung cancer (36). 2,300 common genes were identified between the two platforms used upon downregulation of SRSF1, and a combined 31/40 (77.5%) splicing events were further validated (**Supplementary Table 1**) (36). ATP11C, IQCB1, TUBD1, and PRRC2C were clinically validated and confirmed to be deregulated in primary lung tumors (36). Wang et al. showed that splicing of bridging integrator 1 (Bin1) is under the control of SRSF1 (37). Bin1 is a tumor suppressor involved in DNA damage response, and the loss of Bin1 can confer chemotherapeutic resistance (38). Bin1+12a isoform was determined to abolish its tumor suppressor role in NSCLC and is promoted by SRSF1 overexpression (37). SRSF1 was also found to be a direct target of oncogenic transcription factor MYC in lung carcinomas, and its induction was shown to be responsible for AS of signaling kinase MKNK2 that codes for the eIF4e-kinase Mnk2 and TEAD1 that codes for the transcriptional enhancer factor TEF-1 (33). SRSF1 was shown to be partly responsible for the oncogenic activity of MYC, as SRSF1 knockdown in MYC-transformed cells impaired anchorage-independent growth and proliferation (33).

In neuroendocrine lung tumors, SRSF2 and p-SRSF2 were determined to be overexpressed and were implicated in the control of cell cycle genes such as cyclin E through interaction with and stimulation by E2F1 (39). E2F1 was also found to cooperate with SRSF2 for AS of pro-apoptotic splice variants of c-flip, caspases-8 and -9, and Bcl-x in human lung carcinoma cells (40). In addition, transcriptional activation of SRSF2 *via* E2F1 was shown to be partly responsible for AS of VEGF in favor of the anti-angiogenic isoform (i.e. VEGF_165b_), and was confirmed, *in vivo*, that this splice switch was able to reduce tumor neovascularization (41). Relative to SRSF1 and SRSF2, much less is known about the other SR proteins and their relationship with cancer.

Oncogenic functions of SRSF3, including enhanced proliferation, clonogenicity, migration, and invasion, were found to be partly mediated by its regulation of miR-1908 through NF-κB transactivation, independent of the miR-1908 host gene, FADS1 (42). Mature miR-1908, miR-1908-5p, was subsequently shown to bind to the 3’UTR and inhibit NF-κB inhibitor interacting Ras-like 2 (NKIRAS2), a negative regulator of the NF-κB signaling pathway (42). Perpetually active NF-κB signaling has long been associated with cancer development and progression, thereby linking SRSF3 to oncogenic potential involving NF-κB positive feedback regulation. Global profiling of SRSF3-regulated differential gene expression and splicing events in human osteosarcoma U2OS cells showed that SRSF3 regulates more than 200 targets related to proliferation, cell cycle, cytoskeleton, and RNA splicing (43). In addition, SRSF1 and SRSF3 were found mutually dependent and co-expressed in normal and tumor tissues/cells (43). SRSF3 was also found to significantly regulate the expression of at least 20 miRNAs, many of which are oncogenic or tumor suppressive (43). In human colorectal cancer cells, SRSF4 was found to interact with a novel enhancer on exon 6 of Fas pre-mRNA to stimulate its inclusion, generating the membrane-bound pro-apoptotic isoform (44). In newly diagnosed acute myeloid leukemia (AML) patients, SRSF4 mRNA was found significantly decreased in peripheral blood mononuclear cells (PBMCs) (45). In addition, SRSF4 was positively correlated with caspase 8L expression in AML patients (45). In MCF-7 breast carcinoma cells treated with cisplatin, SRSF4 knockdown abrogated the changes in AS otherwise induced by cisplatin (46). Moreover, SRSF4 knockdown in cisplatin treated cells strongly reduced cell death in the presence of cisplatin, suggesting that SRSF4 plays an important role in chemotherapeutic treatment (46).

SRSF5 and SRSF3 were reported to be overexpressed in oral squamous cell carcinoma (OSCC), and necessary for OSCC cell proliferation, cell cycle progression, and *in vivo* tumor formation (47). Recently, SRSF5-7 were all found to be upregulated in small cell lung cancer (SCLC) and NSCLC tissues, and knockdown of SRSF5-7 in SCLC cell lines showed a significant decrease in proliferation (48). In SCLC cell lines, SRSF6 knockdown showed a significant decrease in invasion and migration (48). In basal and squamous cell carcinomas of the skin and in melanoma, SRSF6 is overexpressed and was shown to act as a proto-oncogene (49). SRSF6 overexpression in transgenic mice induced hyperplasia of sensitized skin and aberrant AS, including that of extracellular-matrix protein tenacin C, which is associated with metastasis and invasion (49). In renal cancer cells, SRSF7 was shown to be regulated by miR-30a-5p and miR-181a-5p, and AS of osteopontin was shown to be under the control of SRSF7 (50). Silencing of SRSF7 was shown greatly reduce cell proliferation in renal cancer cells (50). In lung and renal cancer cell lines, SRSF7 was shown overexpressed and SRSF7 knockdown inhibited proliferation and enhanced apoptosis (51). Moreover, SRSF7 knockdown and overexpression in A549 and BEAS-2B cells, respectively, demonstrated that SRSF7 upregulation impacts the AS of Fas by increasing the skipping of exon 6, promoting the production of the more soluble, pro-survival variant (51). SRSF7 was found overexpressed in colon cancer cell line, and SRSF7 knockdown inhibited growth and blocked cell cycle progression from G1 to S phase (52). In addition, knockdown of SRSF7 induced p21 without any induction of p53, and reduced the levels of phosphorylated CDK2 and pRb along with CDK2 protein (52). The effect of SRSF7 depletion on p21 was attributed to the stabilization of p21 mRNA and protein (52). Overexpression of SRSF9 and SRSF1 were shown to promote accumulation of β-catenin, a key event in Wnt signaling and tumorigenesis, which was necessary for NIH/3T3 cell transformation (53). SRSF9 was further shown to be overexpressed in glioblastoma, colon adenocarcinoma, squamous cell lung carcinoma, and malignant melanoma tissues (53).

In summary, SR proteins demonstrate a vast reach with regards to their splicing profiles across many different cancer types (**Supplementary Table 1**). Their dysregulation in cancer generates transcripts that perpetuate tumor growth by a variety of different molecular mechanisms. Mutations in *cis*-regulatory elements in oncogenes and tumor suppressors and alterations in *trans*-acting splicing factors have clearly demonstrated the importance of AS in carcinogenesis and tumorigenesis.

### SR Protein Phosphorylation and Splicing Regulation in Cancer

Phosphorylation of SR proteins impacts their subcellular localization, their association with the spliceosome complex, and splicing activity (54).SR proteins are phosphorylated at multiple serine residues located in their RS domain, which can range from ~50-300 residues and differ in overall arginine-serine content (55). Some RS domains contain >6 repeats while others contain numerous short stretches of four or fewer dipeptide repeats (55). Multiple kinases are involved in SR protein phosphorylation and include: SR protein kinases (SRPK1-3), cdc2-like kinases (CLK1-4), topoisomerase I (TOP1), PRP4, NEK2, and other well-known kinases such as AKT that are implicated in many key cellular processes (56). A number of studies have been conducted that describe the mechanisms and kinetics of SRPK1’s phosphorylation of SRSF1.

**Figure 3:**
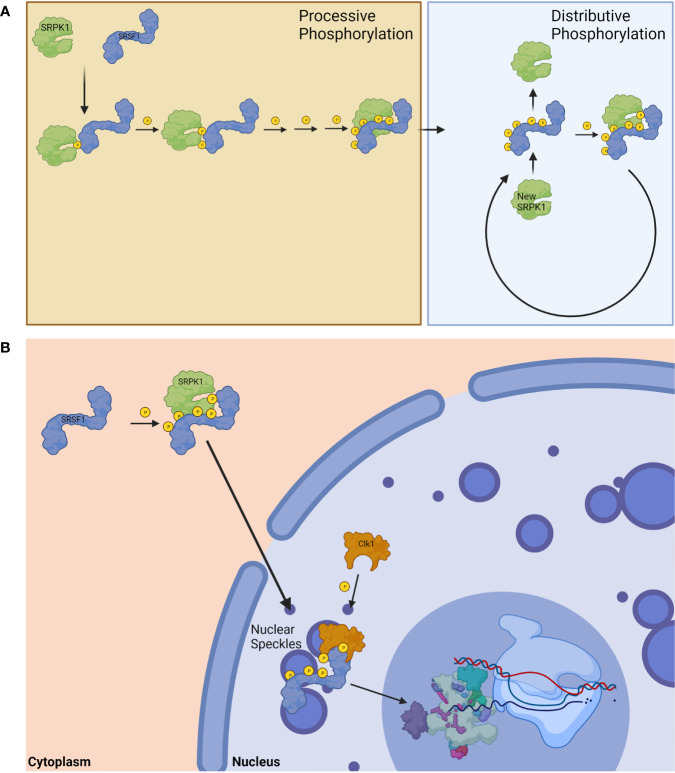
Phosphorylation of SRSF1. **(A)** SRPK1 phosphorylates SRSF1 in a processive fashion until exhausted before switching to distributive phosphorylation. **(B)** The phosphorylation of SRSF1 allows for its translocation from the cytoplasm to the nucleus. Hypophosphorylated nuclear SRSF1 are localized in nuclear speckles until Clk1 phosphorylation releases them for spliceosomal function. Created with BioRender.com.

SRPK1 can phosphorylate up to 12 serine residues in SRSF1 (54). This phosphorylation occurs in a processive fashion, and SRPK1 was shown to phosphorylate 5-8 of the 12 available serine residues in the RS domain before its affinity diminished and shifted to distributive phosphorylation (**Figure 3A**) (54, 57). Contrary to previous thought, it was recently shown that the nonconsensus region in the RS domain of SRSF1 is not silent with regard to phosphorylation by SRPK1, and plays an active role in controlling phosphorylation efficiency (55). This is an important consideration for future studies on phosphorylation of SR proteins by their respective kinases. Hypophosphorylated SR proteins are stored in nuclear speckles until they undergo sufficient phosphorylation (56); sufficient phosphorylation of SR proteins mediated by Clk1 permits for transportation from nuclear speckles, where they can then take part in splicing (**Figure 3B**) (56).

Kinases that target SR proteins are frequently dysregulated in many different cancer types (56). In fact, by using data from TCGA, Czubaty et al. showed that SRPKs, CLKs, and NEK2 are frequently altered in many cancers (56). TOP1 and PRP4 showed moderate elevation across cancer types, and the most pronounced changes for PRP4 were observed in thymoma and kidney chromophobe subtype while the most pronounced changed for TOP1 were observed in lung, stomach and esophagus, and uterine corpus endometrial carcinomas (56). By and large, cancer-related literature concerning SR protein phosphorylation pertains to SPRKs and CLKs.

The value of SRPK1 as a prognostic marker for cancer was recently reviewed by Nikas et al., 2019, and they posit that SRPK1 is indeed a promising prognostic marker for non-small cell lung, breast, prostate, colorectal, stomach, liver, and esophageal cancers based on clinicopathological criteria (58). Less promising is its application as a prognostic marker in non-epithelial cancers including gliomas, germ cell tumors, and retinoblastomas (58). Evidence seems to suggest that SRPK2 plays a similar oncogenic role, albeit not much is known about SRSF2 compared to SRSF1. Most recently, SRPK2 expression was shown to be higher in colorectal cancer tumors compared to nontumor tissues, and was positively associated with tumor differentiation, primary tumor (T) stage, regional lymph nodes (N) stage, and UICC stage (59). The same authors showed that SRPK2 promotes pancreatic cancer progression by downregulating Numb and p53, and this negative regulation also occurred following 5-fluorouracil or cisplatin treatment in colorectal cancer cells [(37, 59). Overexpression also increased cell migration and invasion, and decreased chemosensitivity to 5-fluorouracil or cisplatin (59). The functions of SRPK1-2 in cancer are understood to a much greater extent than SPRK3. The documented role that SRPK3 plays in cancer is limited to a tumor suppressor in rhabdomyosarcoma (60). More specifically, SRPK3 was reported to be downregulated in rhabdomyosarcoma cells, and its expression promoted the splicing of MEF2Cα2 isoform and induced differentiation (60). The MEF2 family of transcription factors regulates many differentiation programs, including myogenesis (60). Worth note is that this study also showed that restoration of SRPK3 expression or MEF2Cα2 inhibited proliferation and anchorage-independent growth of rhabdomyosarcoma cells (60). Therefore, SRPK1 and 2 act as oncogenes, are expressed in the vast majority of cancers, and possess great potential as prognostic markers and possible therapeutic targets. More evidence is needed to reach a conclusion for SRPK3, however, its heart and skeletal muscle tissue-specific expression would suggest that an oncogenic role may be limited (61).

Unlike SRPKs, CLKs phosphorylate serine/threonine as well as tyrosine residues, and do not display region specificity (56). With respect to SRSF1, Clk1 is capable of phosphorylating all serine residues, which was shown to be physiologically relevant (62). Aubol et al., 2018 reported that the ability to mobilize SRSF1 from nuclear speckles to the nucleoplasm is dependent on active Clk1 (63). Analysis of TCGA data showed high induction of Clk1 in gliomas or renal tumors, whereas in bladder urothelial carcinomas and invasive breast cancers, Clk1 expression was decreased (56). For CLK4, it was found that the most pronounced changes were in clear cell renal cell tumors (56). In hepatocellular carcinomas (HCCs), downregulation of Clk1 and upregulation of Clk2 and Clk3 has been correlated to alternative splicing of SLC22A1, a commonly observed event in poor response to sorafenib (64–66). Interestingly, protein phosphatases PP1A and PP2A were also upregulated in HCCs and exhibited a positive correlation with SLC22A1 alternative splicing (66), suggesting that other enzymes affecting SR protein phosphorylation may serve as additional biomarkers and therapeutic targets.

## Therapeutic Targeting of Alternative Splicing

### Spliceosome Inhibitors

A number of small molecule inhibitors that target the spliceosome or its auxiliary proteins are being investigated as new therapeutic approaches for cancer treatment (**Table 1**). While there are currently no U.S. FDA approved small molecule inhibitors or modifiers of spliceosomal proteins for cancer treatment, many naturally-derived compounds being investigated include, but are not limited to, pladienolides, spliceostatins, isoginkgetin, SRPK, and CLK inhibitors [reviewed in (18, 89)]. Due to the widespread attention that has been given to these compounds over recent years, a plethora of synthetic analogs and structurally related compounds have been discovered, and they also display potent antitumor effects and spliceosome inhibition [reviewed in (89)]. The vast majority of small molecule inhibitors have been shown to target splicing factor SF3b, although some display specific activity against different SF3b subunits or spliceosomal associated proteins (SAPs) which include: SF3B1/SAP155, SF3B2/SAP145, SF3B3/SAP130, and PHF5A/SAP14b (89, 90).

**Table 1:** Spliceosome inhibitors.

AS Inhibitors	Assayed cancers	Target proteins	References
Pladienolide analogs	Gastric cancer, Cervical cancer	SF3b	(67–71)
FR901464, Spliceostatin A, and Sudemycin	Cervical cancer, Rhabdomyosarcoma	SF3b	(72–76)
Isoginkgetin	Thyroid cancer, Fibrosarcoma, Breast carcinoma, Melanoma	U4/U5/U6 tri-snRNP	(77–79)
Compound 21b	Bladder cancer, Breast cancer, Monocytic leukemia	CLK1, CLK4, Dyrk1A	(80)
T-025	Breast cancer	CLK1, CLK2, CLK3, CLK4, DYRK1, DYRK1B	(81)
CC-671	Triple-negative breast cancer	CLK2	(82, 83)
SRPIN340	Melanoma, Leukemia	SRPK1, SRPK2	(84–86)
SPHINX	Prostate cancer	SRPK1	(87, 88)

**Figure 4:**
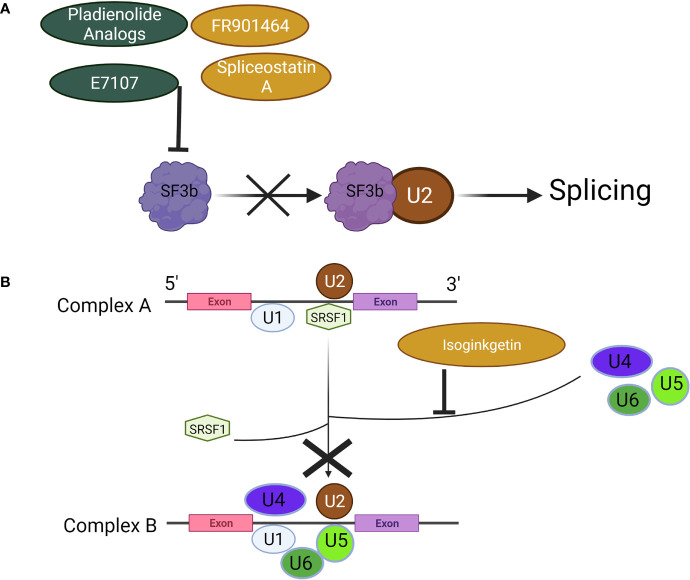
Mechanisms of Spliceosome Inhibitors. **(A)** Pladienolides, FR901464, and their analogs suppress the spliceosome by binding to SF3b to prevent its association with U2, **(B)** Isoginkgetin prevents the recruitment of the U4/U5/U6 tri-snRNP, halting the spliceosome at Complex A. Created with BioRender.com.

Among the more widely studied compounds, the pladienolides analogs (A-G and synthetic analog, E7107) target SF3b, which binds U2 snRNP to disrupt splicing (**Figure 4A**) (67). Antitumor activity of pladienolide B has been demonstrated both *in vivo* and *in vitro*, and in a variety of cancer cell lines including those derived from gastric and cervical cancers and erythroleukemia (68–70). The effects of pladienolide B are attributable to splicing impairment through its impact on genes involved in apoptosis such as p73, whereby pladienolide B increased and decreased the pro- and anti-apoptotic isoforms Tap73 and DNp73, respectively (69). In addition, pladienolide B has been shown to cause cell cycle arrest (70). Despite the encouraging preclinical evidence surround pladienolides, E7107 showed limited efficacy in a phase I clinical trial, and the study was ultimately terminated due to adverse events involving vision loss (71).

FR901464, its methylated derivative spliceostatin A (SSA), and analogous compounds act in a similar manner to pladienolides; their mechanism of action involves inhibition of the SF3b subcomplex (72). The splicing modulatory function of spliceostatins by binding SF3b was originally shown in 2007 along with the accumulation of unspliced pre-mRNA (72). In a series of biological activity studies, Gosh et al., 2014 later showed that both FR901464 and SSA were capable of halting spliceosome assembly at the previously observed A-like complex, resulting in splicing inhibition (73). The primary effect of SF3b inhibition by SSA or related compound, sudemycin, on aberrant splicing in cervical cancer cells as well as rhabdomyosarcoma cells was determined to be exon skipping (91). Exon junctions supporting skipping events were not identified in any annotated isoforms, which raises an important question: what protein functions do these novel transcripts support, absent from those subjected to NMD? While the antitumor activity of SSA or related compounds has yet to be fully mapped, the pro-apoptotic, anti-angiogenic, and cell cycle inhibitory properties resulting from splicing inhibition warrant continued investigation (74–76).

Isoginkgetin was found to inhibit both the major and minor spliceosome by preventing stable recruitment of U4/U5/U6 tri-snRNP, resulting in accumulation of pre-spliceosome A complex (**Figure 4B**) (77). It was subsequently confirmed by observing increased rates of lariat formation using SnapShot-seq that isoginkgetin could block formation of spliceosomal complex B and inhibit splicing (92). In thyroid cancer cells isoginkgetin treatment induced a splice-switch in IL-32, generating IL-32γ, a potent inducer of cell death due to its ability to block IL-8 pro-survival signaling (93). Splicing inhibition achieved with isoginkgetin treatment was also shown to compromise DNA double-strand break repair by downregulating E3 ubiquitin ligase RNF8 and impairing recruitment of DNA damage repair factors, suggesting its utility in cancer treatment when paired with conventional chemotherapeutics that induce DNA damage (94). The antitumor effects of isoginkgetin were demonstrated *in vitro* in fibrosarcoma, breast carcinoma, and melanoma cells by inhibiting invasion (78). Anticancer properties of isoginkgetin involving apoptosis induction and cell cycle arrest were also shown in a variety of additional cancer cell lines, with cervical cancer cells being the most sensitive to treatment (79).

### SRPK and CLK Inhibitors

Targeting SRPKs and CLKs for therapeutic purposes possess great potential considering the malignant potential of aberrantly expressed SR proteins as previously discussed. In 2004 Muraki et al. first described the splicing modulatory effect of Clk1 inhibitor, TG-003, in HeLa cells (95). TG-003 successfully suppressed SR protein phosphorylation and dissociation of nuclear speckles; however, the effects were found to be reversible, and the compound was not toxic (80). Since then, a large number of studies have described Clk inhibitors that display anticancer properties; compound 21b (80), CGP-74514A (96), aminopurvalanol A (96), T-025 (81), and CC-671 (82, 83), among others. Many of these compounds demonstrated particularly promising results based on *in vitro* and *in vivo* studies. Riggs et al., 2017 first showed that compound 23 (i.e. CC-671), a duel Clk2/TTK inhibitor was able to inhibit tumor growth in a TNBC murine xenograft model, and relative to taxotere, the current standard of care for TNBC, showed improved efficacy and was better tolerated (82). Zhu et al. further showed that cell lines representing leukemia, lymphoma, colorectal cancer, head and neck, and bladder cancers were sensitive to CC-671, and confirmed *in vivo* treatment inhibits tumor growth in a TNBC xenograft model along with a patient-derived xenograft model to support further studies for clinical development of this compound (83).

Over the past several years multiple pre-clinical attempts have been made to target SRPKs for therapeutic purposes. Two compounds, in particular, have drawn a moderate degree of attention. Namely, SRPIN340 and SPHINX, originally identified by Fukuhara et al., 2016 and Gammons et al., 2013, respectively (84, 87). SRPIN340 preferentially inhibits SRPK1 and SRPK2, with a higher inhibitory effect on SRPK1, and was shown to reduce human melanoma tumor growth *in vivo*, but not *in vitro*, and was attributable to the regulation of VEGF expression and angiogenesis reduction (85). Siqueira et al. demonstrated that SRPIN340 did display an anti-proliferative effect on leukemia cells and was again attributable to the reduction of pro-angiogenic VEGF_165_ isoform and induction of pro-apoptotic FAS (86). It was recently shown that SRPIN340 was capable of reducing cell migration, invasion, and colony number formation, but did not impact cell viability in the concentration range tested (88). Despite some of the more promising results, SRPIN340 was shown to be poorly absorbed *in vivo*, and local daily subcutaneous injections were required to circumvent its poor pharmacokinetic properties (85). SPHINX was shown to be more selective but similarly potent for SRPK1 compared to SRIN340, was shown to significantly downregulate VEGF_165_ expression, and inhibit tumor growth in an orthotopic mouse model of prostate cancer (87, 97). A few other inhibitors have been documented and include an irreversible inhibitor SRPKIN-1 (98), a series of trifluoromethyl arylamides (88, 99), and a novel chimeric antibody targeting SRPK1 (100), however these compounds have yet to be comprehensively investigated.

## Summary and Perspectives

Splicing is a key process in normal biological functioning, and its dysregulation is strongly linked to cancer development and progression. Small molecule inhibitors and other novel therapeutics (such as antisense oligonucleotides) that target the spliceosome or its auxiliary factors are on the verge of become a new option for cancer therapy. A Phase I first-in-human dose-escalation study is currently being conducted in myelodysplastic syndromes (MDS), acute myeloid leukemia, and chronic myelomonocyte leukemia to evaluate H3B-8800, a small molecule inhibitor of SF3b that potently and preferentially kills spliceosome-mutant tumor cells (101, 102). Data from the ongoing clinical trial was recently published, but did not show any complete or partial responses meeting the international working group criteria (103). That said, red blood cell (RBC) transfusion independence (>56 d) was observed in nine patients who were transfusion dependent at the start of the study (103). Moreover, of the 15 MDS patients with missense SF3B1 mutations, five experienced RBC transfusion independence, which does indicate some degree of clinical improvement and benefit in this subset of patients. The study investigators concluded that further investigation of H3B-8800 in MDS is warranted and suggest exploring alternative dosing schedules. Overall, pre-clinical and clinical data pertaining to spliceosome inhibition, excluding SF3b inhibitors, and novel splicing modulators are greatly lacking, however. SR proteins control vast splicing networks and are commonly altered in a variety of cancers, which suggests that they may be a promising therapeutic target. Splicing networks linked to SR proteins need to also be investigated in order to better understand the extent to which intervention may be useful.

Therapeutic targeting of the spliceosome machinery is an effective approach, however, targeting specific ASEs also possesses unique benefits. Targeting ASEs that are linked to oxidative stress, for instance, may be a lucrative approach. When used in combination with mainline chemotherapeutics that generate high levels of oxidative stress such as anthracyclines, alkylating agents, and platinum coordination complexes, this approach might provide added benefits (104). Aberrant splicing events have a significant impact in promoting resistance to chemotherapy or immunotherapy, which can potentially be overcome by reconfiguring splicing events that favor apoptosis-promoting transcripts, for example (105). In summary, reconfiguring the spliceosome or its accessory components to direct splicing activity or modulate ASEs may be a valuable tool for cancer therapy.

## Author Contributions

Conceptualization, HS; Funding acquisition, HS; Supervision, HS; Writing—original draft, AM; Writing—review & editing, AL, PL, and HS. All authors have read and agreed to the published version of the manuscript.

## Funding

This study was supported by funding from the NIH/NCI, R01 CA217923 and NIH Training Grant 2 T32 ES 7324-21

## Conflict of Interest

The authors declare that the research was conducted in the absence of any commercial or financial relationships that could be construed as a potential conflict of interest.

## Publisher’s Note

All claims expressed in this article are solely those of the authors and do not necessarily represent those of their affiliated organizations, or those of the publisher, the editors and the reviewers. Any product that may be evaluated in this article, or claim that may be made by its manufacturer, is not guaranteed or endorsed by the publisher.
