# Supplementary material for: Therapeutic Targeting of Alternative Splicing: A New Frontier in Cancer Treatment
Source: Front Oncol. 2022 Apr 8;12:868664. doi: 10.3389/fonc.2022.868664 (PMC9027816; doi:10.3389/fonc.2022.868664)
Supplement: Supplementary file 1 [file Table_1.pdf]

Supplemental Table 1: Altered SR Protein Function and Cancer

**SR Protein**	**Associated Cancer Type**	**Expression**	**Associated Genes and Splice Variants***	**SR Protein Associated Biological and Functional Implication**	**References**
SRSF1	Lung	NA	MKNK2	NA	(Das et al., 2012)
			TEAD1	NA	
			MYC	Cell proliferation, cell-cycle progression, and apoptosis	
	Colon	+	NA	NA	(Ajiro et al., 2016)
	Lung				
	Breast				
	Stomach				
	Skin				
	Bladder				
	Liver				
	Cervix				
	Lymphoma cells				
	Acute myeloid leukemia	-	NA	Apoptosis	(Liu et al., 2012)
	Colon carcinoma	NA	beta-catenin	NA	(Fu et al., 2013)
	Colorectal	NA	beta-catenin, cyclin D1	Cell proliferation, colony formation	
	Lung	+	NPRL3, PQBP1, DHPS, CHD3, MYO1B, FN1,WNK1, PPIP5K2, RABL2B, TMPO, MATR3, CXorf26, MYCBP2, ATP11C, SSH1, ABCD4, FIP1L1, TFDP1, MORF4L2, IQCB1, MPV17L, NAGK, RAD51C, MAPT, PAPRBP, ASAP1, EWSR1, TUBD1, SETD5, PRRC2C, USP8	RNA metabolism, cancer and developmental and heriditary disorders, cell division, apoptosis, and proliferation	(de Miguel et al., 2014)
	NSCLC	NA	BIN1 (BIN+12A)	Colony formation, invasion, and apoptosis	(Wang et al., 2019)

Supplemental Table 1: Altered SR Protein Function and Cancer (cont.)

**SR Protein**	**Associated Cancer Type**	**Expression**	**Associated Genes and Splice Variants***	**SR Protein Associated Biological and Functional Implication**	**References**
SRSF2	Neuroendocrine lung carcinoma	+	Cyclin E	Cell cycle progression	(Edmond et al., 2013)
			E2F1	Cell cycle progression, proliferation, apoptosis	
			p45^{SKP2}	Cell cycle progression	
	Lung	NA	c-FLIP (c-FLIP$_{short}$/c-FLIP$_{long}$)	Apoptosis	(Merdzhanova et al., 2008)
			Caspase 8 (8L/8a)	Apoptosis	
			Caspase 9 (9a/9b)	Apoptosis	
			Bcl-x (x$_L$/x$_S$)	Apoptosis	
		NA	VEGF (165b)	Angiogenesis	(Merdzhanova et al., 2010)
SRSF3	Osteosarcoma	NA	NF-kB	Proliferation, clonogenicity, migration, and invasion	(Kim et al., 2017)
			NKIRAS2	Proliferation, clonogenicity, migration, and invasion	
			TAK1	Proliferation, clonogenicity, migration, and invasion	
			FADS1 (miR-1908 host gene, include this?)	Proliferation, clonogenicity, migration, and invasion	
			ERRFI1, ANXA1, TGFB2, PUS3, PKP4, KIF23, EP300, CLINT1, CHK1, SMC2, CKLF, MAP4K4, MBNL1, MELK, DDX5, PABPC1, SP1, SRSF1	Proliferation, cell cycle, cytoskeleton, and RNA splicing	(Ajiro et al., 2016)
	Cervical (cancer tissues v. adjacent normal overexpressed, hela cells for gene and variant expression)	+	ERRFI1, ANXA1, TGFB2, PUS3, PKP4, KIF23, EP300, CLINT1, CHK1, SMC2, CKLF, MAP4K4, MBNL1, MELK, DDX5, PABPC1, SP1, SRSF1	Proliferation, cell cycle, cytoskeleton, and RNA splicing	
	Colon	+	NA	NA	
	Lung				
	Breast				

Supplemental Table 1: Altered SR Protein Function and Cancer (cont.)

SR Protein	Associated Cancer Type	Expression	Associated Genes and Splice Variants*	SR Protein Associated Biological and Functional Implication	References
SRSF3	Stomach				(Ajiro et al., 2016)
	Skin				
	Bladder				
	Liver				
	Lymphoma cells				
	Acute myeloid leukemia	-	Caspase 8 (8L/8a)	Apoptosis	(Liu et al., 2012)
	Oral Squamous Cell Carcinoma	+	SRSF5	Proliferation	(Yang et al., 2018)
SRSF4	Colorectal	NA	FAS (exon 6 inclusion)	Apoptosis	(Jang et al., 2018)
	Acute myeloid leukemia	-	Caspase 8 (8L)	Apoptosis	(Liu et al., 2012)
	Breast carcinoma	NA	MDM2, HNRNPDL, AMZ2	Cell viability and Apoptosis	(Gabriel et al., 2015)
SRSF5	Oral Squamous Cell Carcinoma	+	SRSF5 (autoregulation), SRSF3, MCM2, MCM4	Proliferation, tumorigenesis, cell cycle progression	(Yang et al., 2018)
	Lung	+		Proliferation	(Kim et al., 2016)
SRSF6	Skin (pre-cancerous; epidermal hyperplasia)	NA	Keratin 6, Keratin 16, Tnf, Il1b, Cxcl2, Ccl3, Tenacin c	Miotic cell cycle, response to wounding, epidermal differentiation, cell proliferation, wound healing	(Jensen et al., 2014)
	Basal Cell Carcinoma	+	NA	NA	(Jensen et al., 2014)
	Squamous Cell Carcinoma				
	Melanoma	+	Tenacin c (full length)	NA	
	Lung	+	NA	Proliferation, migration, invasion	(Kim et al., 2016)
SRSF7	Lung	+	NA	Proliferation	(Kim et al., 2016)
	Renal cancer	NA	Spp1 (Spp1-c/Spp1-b)	Proliferation	(Boguslawska et al., 2016)

Supplemental Table 1: Altered SR Protein Function and Cancer (cont.)

SR Protein	Associated Cancer Type	Expression	Associated Genes and Splice Variants*	SR Protein Associated Biological and Functional Implication	References
SRSF7	Colon	+	NA	Apoptosis, cell proliferation	(Fu and Wang, 2018)
	Lung	+	Fas (exon 6 exclusion)	Apoptosis, cell proliferation	(Fu and Wang, 2018)
	Colon	+	p21, cdk2, Rb, cdkn1a	Cell cycle, proliferation	(Saijo et al., 2016)
SRSF9	Glioblastoma	+	NA	NA	(Fu et al., 2013)
	Colon Adenocarcinoma				
	Squamous Cell Lung Carcinoma				
	Malignant Melanoma				
	Colon carcinoma	NA	beta-catenin	NA	(Fu et al., 2013)
	Colorectal	NA	beta-catenin, cyclin D1	Cell proliferation, colony formation	

+/- increased (+) or decreased (-) expression

NA - Not addressed in study

* Indicates validated genes and/or splice variants
